# Spatiotemporal Variation of Burnt Area Detected from High-Resolution Sentinel-2 Observation During the Post-Monsoon Fire Seasons of 2022–2024 in Punjab, India

**DOI:** 10.3390/s25175588

**Published:** 2025-09-07

**Authors:** Ardhi Adhary Arbain, Ryoichi Imasu

**Affiliations:** 1 Research Center for Climate and Atmosphere, Research Organization for Earth Sciences and Maritime, National Research and Innovation Agency, Serpong, Tangerang Selatan 15314, Indonesia; 2 Atmosphere and Ocean Research Institute, The University of Tokyo, 5-1-5 Kashiwanoha, Kashiwa 277-8564, Chiba, Japan; imasu@aori.u-tokyo.ac.jp

**Keywords:** burn severity, burnt area detection, dNBR, fire radiative power, Google Earth Engine, MODIS, Normalized Burn Ratio, PM_2.5_ emission, Sentinel-2

## Abstract

**Highlights:**

**What are the main findings?**

- High-resolution Sentinel-2 observation captures spatiotemporal variability of the fine-scale burnt areas in Punjab, which are often missed by coarser-resolution observation modes such as MODIS.
- PM_2.5_ emissions derived from Sentinel-2 observation are much higher than those reported by the EDGAR v.8.1 global inventory.

**What is the implication of the main finding?**

- More accurate burnt area (BA) detection and PM_2.5_ emission estimation provide considerable improvement over coarse-resolution inventories to support better air quality modeling and monitoring.
- The discrepancy in PM_2.5_ emission estimation underscores the need for observation-driven monitoring systems, rather than the conventional statistics, to support the fire mitigation strategy and to strengthen policy responses to seasonal biomass burning.

**Abstract:**

Underestimation of PM_2.5_ emissions from the agricultural sector persists as a major difficulty for air quality studies, partly because of underutilization of high-resolution observation platforms for constructing a global emissions inventory. Coarse-resolution products used for such purposes often miss fine-scale burnt areas created by stubble-burning practices, which are primary sources of agricultural PM_2.5_ emissions. For this study, we used the high-resolution Sentinel-2 observations to examine the spatiotemporal variability of burnt areas in Punjab, a major hotspot of agricultural burning in India, during the post-monsoon fire season (October–December) in 2022–2024. The results highlight the Sentinel-2 capability of detecting more than 34,000 km^2^ of burnt areas (approx. 68% of Punjab’s total area) as opposed to the less than 7000 km^2^ (approx. 12% of Punjab’s total area) detected by MODIS. The study also reveals, in unprecedented detail, multi-annual spatial and temporal shifting of burning events from northern to central and southern Punjab. This detection discrepancy has led to marked disparities in estimated monthly emissions, with approximately 217.3 million tons of PM_2.5_ emitted in October 2022 compared to 8.7 million tons found by EDGAR v.8.1. This underscores higher-resolution observation systems intended to support construction of a global PM_2.5_ emissions inventory.

## 1. Introduction

Stubble burning is a practice of setting fire to the remaining straw stubble in a cultivated field after the grain or main product has been harvested. Although outcomes are similar, stubble burning is differentiated from wildfires because of the intentional nature of the former and its primary scope of application to the agricultural sector. Despite its benefits as a quick, cheap, and efficient method to prepare soil beds for later crops, stubble burning has been banned in many countries around the globe for decades. In fact, this practice has been cited as a major cause of air pollution and as a driver of climate change by releasing huge quantities of greenhouse gases to the atmosphere [1,2].

For the last two decades, stubble burning in Punjab has emerged as a major environmental issue in India. For instance, post-harvest stubble burning in November engenders nearly 43% aerosol loading over the populous Indo-Gangetic Plain [3]. A recent study revealed that, based on annual particulate matter 2.5 (PM_2.5_) rankings by city, 35 of the top 50 most polluted cities in the world are in India [4]. Most states, and 76.8% of the population, are exposed to an annual population-weighted mean PM_2.5_ greater than 40 μg/m^3^, which is the limit recommended by the Indian National Ambient Air Quality Standards [5,6]. Air pollution, which has affected the health of Indian residents, has increased mortality and morbidity rates nationwide. In 2017, 1.24 million deaths, one-eighth of all deaths recorded in India, were attributed to air pollution, approximately half of which were people younger than 70 years old [5].

For decades, satellite-based remote sensing has been used as a powerful tool for monitoring and detecting physical characteristics over the land, air, and ocean on regional and global scales [7,8,9,10]. Remote sensing has been used specifically for mapping and planning [11,12,13], weather monitoring [14], and change detection [15,16]. Utilization of remote sensing has proven useful for assessing effects of anthropogenic intervention and landscape changes on the environment, such as those to natural vegetation and hydrological ecosystems [17]. Another popular application of remote sensing technology for change detection is for the monitoring and forecasting of wildfires. Various indices, such as Normalized Difference Vegetation Index (NDVI) and Normalized Difference Water Index (NDWI), have been developed to evaluate the relation between remote-sensing-derived variables and fire danger-related indicators [18]. Vast coverage and data availability of remote sensing observation can be combined further with in situ observation or static datasets to improve fire forecasting system performance [19]. These sophisticated systems can be improved even further by combining them with state-of-the-art technology such as machine learning and internet of things (IoT) [20,21]. It is noteworthy, however, that most fire monitoring and forecasting systems are using remote sensing observation in the moderate range and coarse spatial resolution.

The Moderate Resolution Imaging Spectrometer (MODIS) and Visible Infrared Imaging Radiometer Suite (VIIRS) have been used particularly and widely for fire monitoring systems, especially to detect active fire hotspots (FHS) and burnt areas, which are the main sources of air pollution [7,8,22,23]. Subsequently, the FHS information from these observations is used to construct global fire emission inventories, which are fundamentally important for models of atmospheric composition to reconstruct and project the effects of biomass burning on the air quality, public health, ecosystem dynamics, climate, and land–atmosphere exchanges [24]. The coarse resolution of MODIS and VIIRS products, however, renders them ineffective for the detection of fire hotspots smaller than their grid cells, which engenders underestimation of the total burnt area and erroneous simulation of aerosol emissions over the target region [24,25].

As described herein, we demonstrated the capabilities of Sentinel-2 [26] observations to examine the spatiotemporal variability of stubble-burning activity in the state of Punjab, northern India, during post-monsoon fire seasons across the three consecutive years of 2022–2024. Numerous studies have been undertaken during the past 15 years to monitor burnt areas in India. Research using data from advanced wide field sensor (AWiFS) observations has been reported for the Punjab region in 2005, yielding an estimation of the extent of burnt areas and greenhouse gas (GHG) emissions from crop residue burning [27]. Similar attempts have been undertaken using fine-resolution images of LISS III and Landsat 8 Operational Land Imager (OLI) satellites in 2007 and 2014–2018 [28,29]. Using data from these fine-resolution observations enables burnt area mapping in high detail and with high accuracy, but cloud obstruction and low revisiting frequencies of these satellites have posed difficulties for temporal analysis and prediction, thereby prompting scholars to use data from observations with coarser spatial resolution but with higher revisit frequency [30].

In contrast to MODIS and VIIRS, which were typically used by global fire emission inventories, the high-resolution and five-day revisiting frequency of Sentinel-2 (with two satellites) enable the use of composite images to detect burnt scars left after stubble-burning activities, to reduce the obscuring effects of clouds and aerosols, and to detect surface changes with greater detail and higher accuracy. Moreover, the versatility of Sentinel-2 observations has been extended recently by the incorporation of machine learning technology to improve the accuracy of burnt area assessment over Punjab [31]. For this study, we opt to use Sentinel-2 observation for burnt area detection using a consistent, interpretable, and operationally efficient method of the differenced Normalized Burn Ratio (dNBR), which has proven reliable for capturing areas scarred by burning over various land surfaces [32]. Unlike machine learning approaches, which often require large, spatially diverse training datasets and complex interpretability, the dNBR method offers a physically meaningful index that can be applied easily for large areas and multiple time periods, making it suitable for examining the temporal dynamics of stubble burning to support regional fire management efforts.

Furthermore, we implemented this study using Google Earth Engine (GEE) v.1.5.12, a state-of-the-art cloud computing platform, which provides access to an extensive geospatial data catalog along with on-the-fly computation for analyzing dynamic changes in agriculture, natural resources, and climate [33]. The GEE allows users to collaborate using data, algorithms, and visualization, thereby enabling this study to be implemented easily in the future, either for research or operational purposes (e.g., monitoring and prediction).

## 2. Materials and Methods

### 2.1. Area of Interest

**Figure 1:**
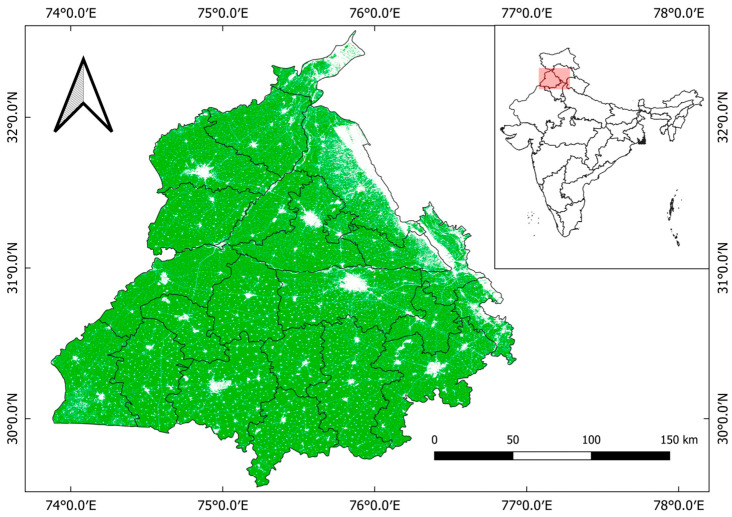
Map of the study area in Punjab, India, showing district boundaries and cropland distribution. Cropland areas are highlighted in green. (*Data source: ESA WorldCover 2021; projection: EPSG:4326*).

This study specifically examines the state of Punjab, India, with a total area of 50,225 km^2^ and located between 29°33′ N and 32°30′ N, 73°53′ E and 76°54′ E, as shown in Figure 1. This region is well known as a main producer of wheat and rice for the country. Moreover, it has been reported repeatedly in recent years as a major hotspot for stubble burning, a practice commonly adopted by farmers to clear rice residue quickly in preparation for the subsequent wheat crop [2,34]. The agricultural calendar in Punjab typically involves sowing rice (Kharif crop) in May–June, with harvesting from October to mid-November. Immediately thereafter, wheat (Rabi crop) is sown for the winter, which extends from November through April; it is harvested during April–May. This tight cropping cycle allows only a brief window of 10–15 days for field preparation between the rice harvest and wheat sowing. Because of the time and economic constraints, burning of crop residues remains a widely adopted method for land clearing [35,36]. Based on this fact, our study targets the post-monsoon fire season (October–December) during three consecutive years: 2022, 2023, and 2024. That period typically coincides with intense stubble-burning activity.

### 2.2. Data

**Figure 2:**
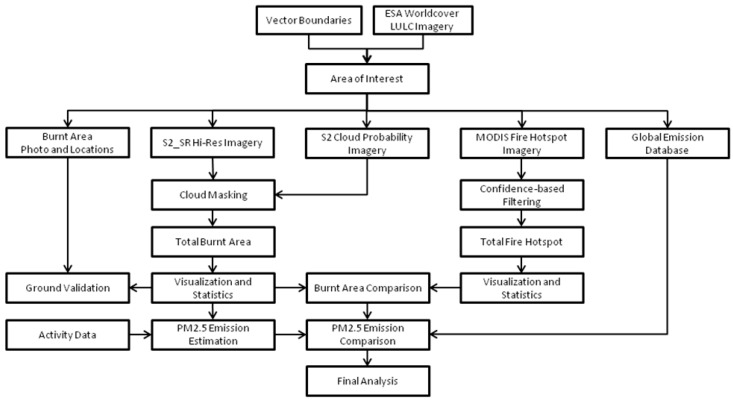
Workflow for burnt area detection in Punjab.

The workflow of this study is presented in greater detail in Figure 2. To investigate the spatial and temporal characteristics of burnt areas in Punjab, we used high-resolution satellite imagery and conducted a comparative analysis using multiple datasets that are freely available on the Google Earth Engine Data Catalog (https://developers.google.com/earth-engine/datasets, accessed on 1 May 2025). Burnt area (BA) detection in this study was conducted using Sentinel-2 Surface Reflectance (S2 SR) imagery acquired from the COPERNICUS/S2_SR_HARMONIZED collection. The dataset provides atmospherically corrected surface reflectance values at a spatial resolution of 10–20 m across 13 spectral bands. Images of October–December during 2022–2024 are selected corresponding to the post-monsoon fire season in Punjab. Cloud-contaminated pixels were removed using a combination of the Scene Classification Layer (SCL) and the Sentinel-2 cloud probability dataset (COPERNICUS/S2_CLOUD_PROBABILITY). Specifically, pixels which are classified as cloud, cirrus, or shadow in the SCL were masked out. Then an additional threshold of cloud probability > 70% was applied to exclude high-confidence cloudy pixels.

To ensure that calculations were restricted to agricultural areas, the ESA WorldCover 10 m 2021 land cover (ESA/WorldCover/v200/2021) dataset was used to mask non-cropland classes. Only pixels classified as cropland (class value 40) were retained; all other land cover types were excluded from the analysis.

To support the analysis and to provide a broader perspective, we also used MODIS active fire data from both the MOD14A1 (Terra) and MYD14A1 (Aqua) products, which are available on GEE’s MODIS/061/MOD14A1 and MODIS/061/MYD14A1 collections. These products provide fire pixel detections at 1 km spatial resolution based on thermal anomalies. Fire detection data from both satellites were aggregated over the same two-week periods for comparison with Sentinel-2-derived BA maps.

As a qualitative validation step, we used geo-referenced field photographs of confirmed locations that had been burnt in Ludhiana district, Punjab. The photographs, which had been taken on 30 October and 6 November 2022, were matched with the corresponding Sentinel-2 BA maps to confirm the BA detection accuracy visually. Although field-based observations were only available for part of the study period, they provide valuable evidence supporting the spatial validity of the spectral-based approach used for this study.

To assess the broader atmospheric effects of burning activity, PM_2.5_ emissions estimated from Sentinel-2-derived burnt areas were compared with anthropogenic PM_2.5_ data from the Emissions Database for Global Atmospheric Research (EDGAR) v.8.1 (https://edgar.jrc.ec.europa.eu/dataset_ap81, accessed on 1 June 2025) global emission inventory, which provides gridded emissions from 1970 to 2022 at a spatial resolution of 0.1° × 0.1°. EDGAR offers sector-specific and total PM_2.5_ emissions at a global scale, which facilitates direct comparison between satellite-based fire-related estimates and reported anthropogenic emissions over Punjab during the most recent overlapping year.

### 2.3. Identification of Burnt Areas

#### 2.3.1. Sentinel-2 dNBR

To identify BA over northern India, the Normalized Burn Ratio (NBR) has been used. The NBR index has also been used for several studies [25,29,37]. Reportedly, as an excellent method for assessing burnt areas [38,39], this index can map burnt areas effectively for many different landscapes worldwide using its near-infrared (NIR) and short-wave infrared (SWIR) bands. The burnt area extent can be estimated by exploiting the distinction between spectral responses of healthy vegetation. A high NBR value represents healthy vegetation, whereas a low value represents bare ground and areas affected by recent burning. The NBR index is calculated using the following equation

(1) $$NBR=\frac{NIR-SWIR}{NIR+SWIR}.$$

The NIR band of Sentinel-2 (Band 8) includes wavelengths of 835.1 nm (Sentinel-2A) and 833 nm (Sentinel-2B). Sentinel-2 provides two SWIR bands (Band 11 and 12) with identical resolution (20 m). However, for this study, we chose Band 12 of Sentinel-2, which has longer wavelengths (2202.4 nm and 2185.7 nm) than Band 11 (1613.7 nm and 1610.4 nm), to achieve better spectral response for the burnt areas. The difference between prefire and postfire NBR obtained from the images is used to calculate the *delta* NBR (dNBR or ∆NBR). A higher value of dNBR represents more severe damage, whereas areas with negative dNBR values might denote regrowth following a fire. The formula used to calculate dNBR is presented below

(2) dNBR=Prefire NBR−Postfire NBR.

A time series of dNBR values can be generated by calculating the NBR indices for certain prefire and postfire periods, for instance, at weekly or monthly intervals. For an interval *i*, the formula can be rewritten as presented below

(3) $${dNBR}_{i}={NBR}_{i-1}-{NBR}_{i}.$$

The tight schedule between the harvest and sowing periods in northern India, especially in Punjab, engenders rapid changes over the land surface. For that reason, using an interval longer than two weeks might hinder burnt area detection. However, using a shorter time interval might engender more missing scenes in the composites. To avoid such outcomes, we examined the burn severity variation during a two-week interval: days 1–15 and 16 to the end of the month, resulting in intervals of 16–31 for October and December and 16–30 for November. A threshold of dNBR ≥ 0.1 was applied to classify pixels as burnt [32]. For this study, we exclusively emphasize burnt areas and therefore consider dNBR values below 0.1 as unburnt, without accounting for post-fire vegetation growth. The burn severity values calculated from dNBR for this study are presented in Table 1.

#### 2.3.2. MODIS Fire Hotspot

Hotspot data were retrieved by filtering daily MODIS fire detections to include only pixels with a “nominal” confidence level, based on the confidence flag provided in the MOD14A1 (Terra) and MYD14A1 (Aqua) products. The “nominal” confidence class offers a practical balance: it includes detections that are reasonably reliable without excluding moderate-intensity fires; such detections are common in cropland burning scenarios such as those observed in Punjab. Using only the nominal category, we reduced false positives while preserving a representative dataset of actual fire activity that is suitable for comparison with Sentinel-2-derived burnt area patterns.

The data were constrained spatially to the Punjab region and were temporally limited to the October–December period during 2022–2024, which aligns with the S2 SR observation window. Detections from both Terra and Aqua were then merged and aggregated into two-week intervals, corresponding to the compositing periods used for the Sentinel-2 analysis. The total BA derived from MODIS was then estimated by multiplying the number of active fire pixels by the nominal spatial resolution of 1 km^2^ per pixel, while assuming that each instance of fire detection represents at least one square kilometer of BA. This estimate, which provides an upper-bound approximation of fire-affected surfaces, was used to compare temporal patterns and magnitudes of BA between MODIS and Sentinel-2 datasets.

### 2.4. Estimated PM_2.5_ Emissions

To estimate PM_2.5_ emissions from agricultural burning, we applied the emission factor (EF) approach, which calculates emissions as the products of BA, available fuel load (biomass), and pollutant-specific emissions factors. The total emissions were computed as presented below

(4) $${PM}_{2.5}={BA}_{{km}^{2}}\times B\times EF\times 1000.$$

In that equation, BA_km_^2^ denotes the Sentinel-2-derived BA in square kilometers, *B* represents the effective dry biomass loading in kilograms/m^2^, and *EF* is the emission factor in kilograms of PM_2.5_ per kilogram of dry biomass that is burnt.

Because rice is the dominant crop in the region, parameter values were selected accordingly. The dry biomass loading for rice residues is typically 4.2–8.0 t/ha (0.42–0.80 kg/m^2^), depending on local agronomic practices [40,41]. A midpoint value of 0.61 kg/m^2^ was adopted. With an assumed burning efficiency of 80%, the effective fuel load was set as 0.49 kg/m^2^. The EF for PM_2.5_ was taken as 9.1 g/kg (0.0091 kg/kg), which is consistent with measurements for open-field rice straw burning in Southeast Asia [42,43]. This EF value lies within the reported range of 4.2–20.7 g/kg for rice residue combustion. Because the burnt area was derived at a two-week temporal resolution, biweekly PM_2.5_ emissions estimation was adopted. This estimation allowed us to construct a two-weekly time series of emissions, enabling more detailed assessment of emission dynamics during the peak burning season (October–December).

To assess the representativeness of PM_2.5_ emissions estimated from Sentinel-2-derived burnt areas, we compared them with anthropogenic emissions from the EDGAR v.8.1. We specifically used the agricultural waste burning sector of the EDGAR dataset, which provides globally consistent, gridded annual emissions of greenhouse gases and air pollutants, including PM_2.5_. EDGAR emissions are derived using the EF approach, by which total emissions are calculated as the product of activity data (AD) and sector-specific EFs, following guidelines from the Intergovernmental Panel on Climate Change (IPCC). These calculations are harmonized across countries using internationally reported data and are allocated spatially using geospatial proxies.

For comparison with our Sentinel-2 results, we extracted EDGAR PM_2.5_ emission data for the Punjab region for 2022, which is the latest year currently available. Although EDGAR emissions represent anthropogenic fire activity at national scale, they provide a useful reference for evaluating the magnitude and spatial pattern of PM_2.5_ released from agricultural burning, as inferred independently from high-resolution satellite-derived burnt area data.

## 3. Results

### 3.1. Burnt Area Detection from Sentinel-2

**Figure 3:**
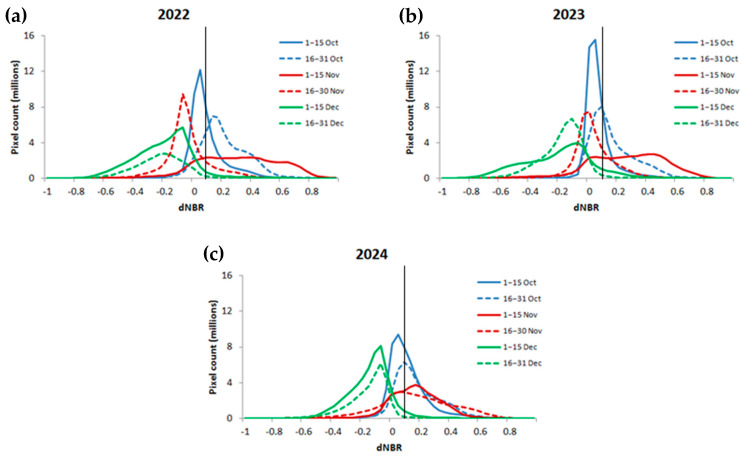
Comparison of bi-weekly dNBR histograms across Punjab for 2022–2024 ((**a**), (**b**), and (**c**), respectively). Each curve represents the distribution of newly detected burnt pixels during the corresponding two-week interval, computed with a bin width of 0.04 using consistent bin edges. Histograms are displayed as line plots for clarity. The vertical black line at a dNBR value of 0.1 denotes the threshold used to classify burnt pixels.

Figure 3 portrays histograms constructed from two-weekly dNBR values across Punjab during October–December in 2022–2024. Each histogram reveals a dominant unimodal pattern of dNBR values for each period of observation. Across all three years, the October 1–15 curves have a peak near zero, suggesting minimal or no burning. However, in later periods (November–December), the histograms flatten and shift rightward with increased dNBR values, indicating the emergence of burn signals. The histograms for 2022 and 2023 have more pronounced right-side tails in November–December curves compared to 2024, which suggests a higher frequency of moderate to high burn severity during those years. The 2024 curves (especially 16–30 November) show a less steep rise beyond 0.1, which might indicate lower fire intensity or coverage. By contrast, the 16–30 November 2024 curve represents the only period among those of the same observation time during which dNBR primarily shifts towards values larger than the 0.1 threshold, indicating a prolongation of severe burn signals compared to earlier years. Figure 3 also shows that the 0.1 dNBR threshold line intersects the histograms near the inflection point where the frequency starts declining, which indicates that the threshold is empirically reasonable to separate unburnt and burnt areas.

**Figure 4:**
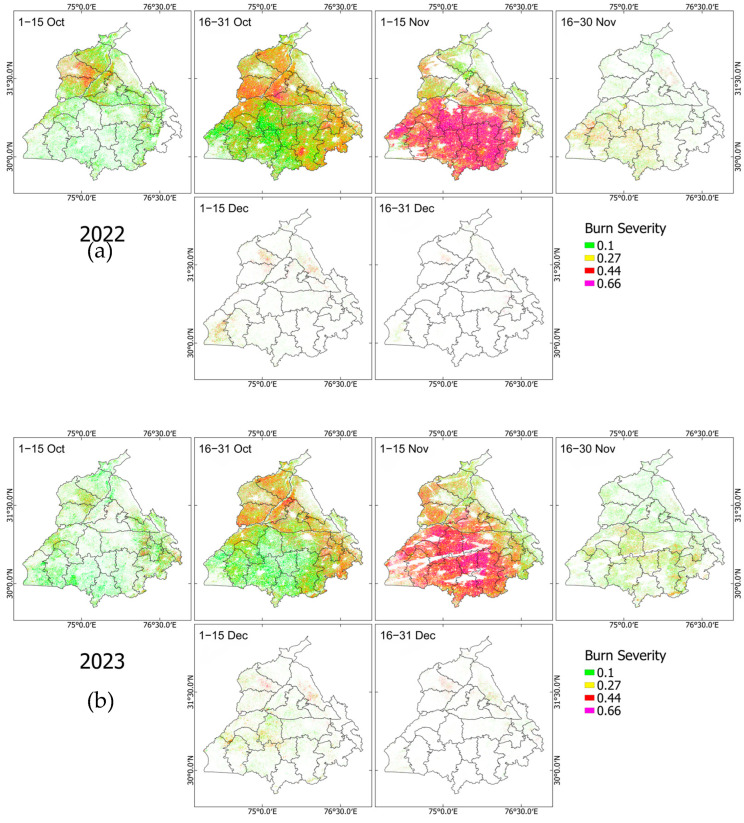

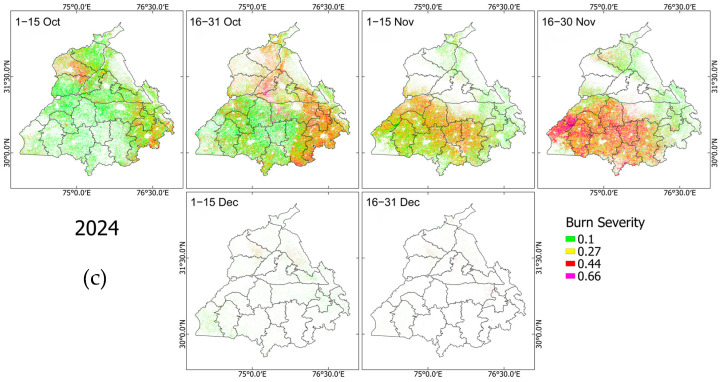
Burn severity maps of Punjab for October–December from 2022 (**a**), 2023 (**b**), and 2024 (**c**). White areas represent unburnt regions, non-cropland areas, and regions omitted because of cloud masking.

Figure 4a–c show the BA maps derived from the dNBR values across Punjab for each two-week period of observations. Across all three years, fire activity consistently begins in early October with scattered low-severity burns (dNBR approx. 0.1, green), primarily in the northern and central parts of the state. Fire extent and severity then increase considerably in late October (16–31 October), followed by a peak in both spatial coverage and severity during the first half of November (1–15 November). During this peak period, large areas in southern and eastern Punjab exhibit high burn severity, with numerous pixels exceeding dNBR values of 0.44 (red) and 0.66 (magenta), indicating intense burning. Post-mid-November marks the decline of new fire activity across the region. The 16–30 November and both December intervals show marked reductions in burnt area, with only sparse, isolated pixels detected, often in the central or western districts.

Although the overall spatial pattern remains broadly consistent across years, a subtle westward shift in burn hotspots is observed in 2024 during the latter half of November. Similarly to the histogram, the BA map during this period also confirms the prolonged burning event in southern Punjab at the end of November 2024. The recurrence of intense burn activity in the same districts over multiple years, which progresses from northern to southern Punjab, suggests spatial persistence of agricultural burning practices, which might be influenced by crop harvesting cycles, land use patterns, mechanization, or even air pollution management policy in the state. This finding clearly indicates the period of 1–15 November as the crucially important window for both the highest fire activity and the most severe burns in the region.

**Figure 5:**
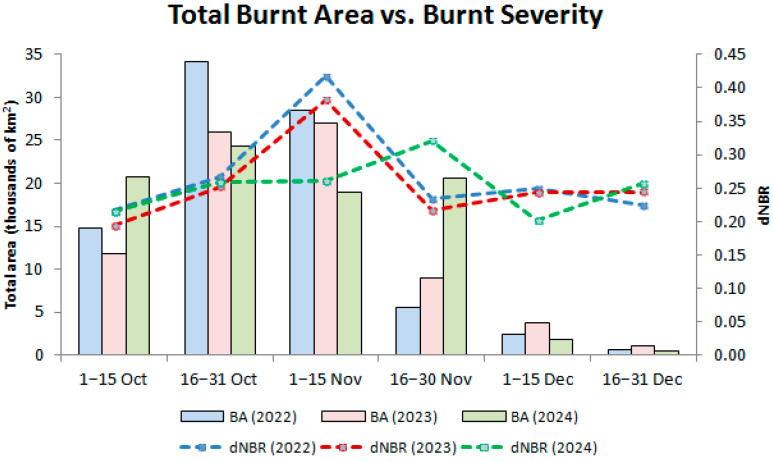
Total burnt areas (BAs), shown as colored bar charts, and mean burn severity (dNBR), shown as colored dashed lines, across Punjab during October–December in 2022–2024.

The total BA and mean burn severity (dNBR) calculated across Punjab during the study period are presented in Figure 5. A distinct peak in both total BA and burn severity occurs during 1–15 November in 2022–2024, confirming this period as the most active phase of stubble burning in the region. In 2022, this period shows the highest BA, exceeding 35,000 km^2^, with a mean dNBR exceeding 0.40, which indicates widespread and severe fires. By comparison, both 2023 and 2024 had slightly lower total BA during this peak period, with values just under 30,000 km^2^, but still maintaining high burn severity (~0.38 for 2023 and ~0.30 for 2024), suggesting continued burn severity.

The pre-peak phase (1–31 October) shows a steady BA increase across all years, with 2022 again showing the largest extent in the late October interval. Mean dNBR values during this period were moderate (from approx. 0.20 to approx. 0.30), suggesting mostly low to moderate severity burns. It is noteworthy that, in 2024, the total BA during October was lower than in preceding years, but the burn severity was more or less stable, possibly indicating fewer but more intense burns. A sharp decline in all BAs occurred in all years after mid-November. The intervals of 16 November–31 December show progressively decreasing fire activity, with total BAs dropping to below 10,000 km^2^. However, the mean burn severity remained around 0.22–0.25, implying that although the number of fires decreases, the severity of individual burns does not diminish drastically.

Overall, Figure 5 reinforces the temporal pattern observed in the BA maps: a buildup of fire activity in October, a pronounced peak in early November, and a rapid decline thereafter. It also highlights 2022 as the most active year in terms of both extent and intensity, whereas 2024 exhibits slightly lower peak values with a more gradual and prolonged severity profile.

### 3.2. Ground Validation

**Figure 6:**
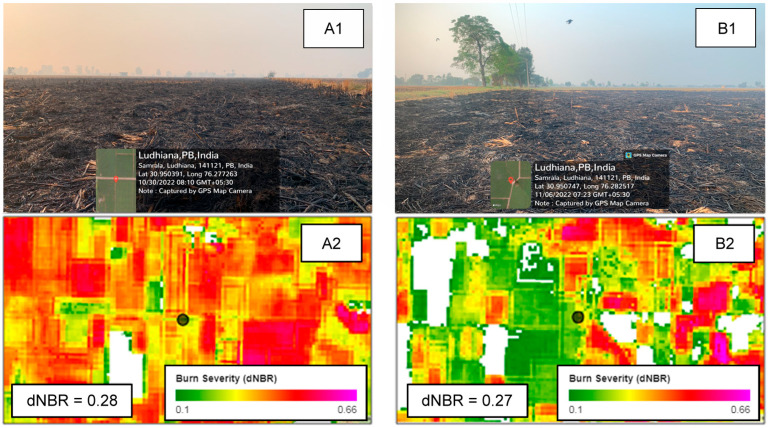
Ground photographs of post-fire fields at two locations in Ludhiana, Punjab, taken on 30 October 2022 (**A1**) and 6 November 2022 (**B1**). BA maps of the corresponding area are presented in the bottom panels ((**A2**) and (**B2**), respectively), with each exact location denoted as a black circle. The burn severity value of each location is shown at the bottom left corner of the BA maps. White areas in BA maps represent unburnt regions, non-cropland areas, and regions omitted because of cloud masking.

To assess the reliability of burnt area detection derived from Sentinel-2 observation, we conducted a visual validation of known burnt fields across Punjab. Several ground photographs were taken in the district of Ludhiana, Punjab. However, only two representative examples are included in this manuscript (Figure 6) to illustrate typical post-burnt field conditions and their correspondence with the Sentinel-2-derived burn severity maps. The first validation point is located at the coordinates of 30.95039° N, 76.277263° E, whereas the second one is located at 30.950747° N, 76.282517° E. The photographs were taken, respectively, on 30 October and 6 November 2022.

The ground photographs (A1 and B1) clearly show post-fire agricultural fields with charred stubble, darkened soil, and an absence of crops, which strongly indicate recent burning. The corresponding dNBR values (0.28 in A2 and 0.27 in B2) are within the moderate burn severity range based on typical dNBR classification thresholds. These photographs also reveal clear boundaries between burnt and unburnt fields, which are detected successfully by Sentinel-2. These characteristics accurately demonstrate the capabilities of Sentinel-2 data to detect and quantify burn severity at fine spatial scales.

### 3.3. Comparison with MODIS Burnt Area Products

**Figure 7:**
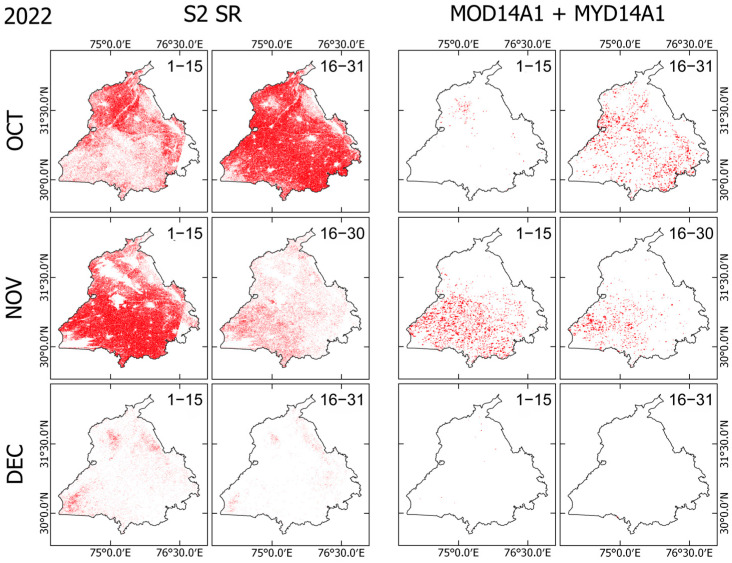
Comparison of newly detected burnt areas (BAs) from Sentinel-2 Surface Reflectance (S2 SR; columns 1–2) and MODIS (MOD14A1 + MYD14A1; columns 3–4) in 2022. Each row corresponds to a month, with biweekly periods shown in the top-right corner of each panel. For each interval, only pixels identified as newly burnt (i.e., not classified as burnt in earlier periods) are mapped, ensuring no double counting across consecutive time steps.

**Figure 8:**
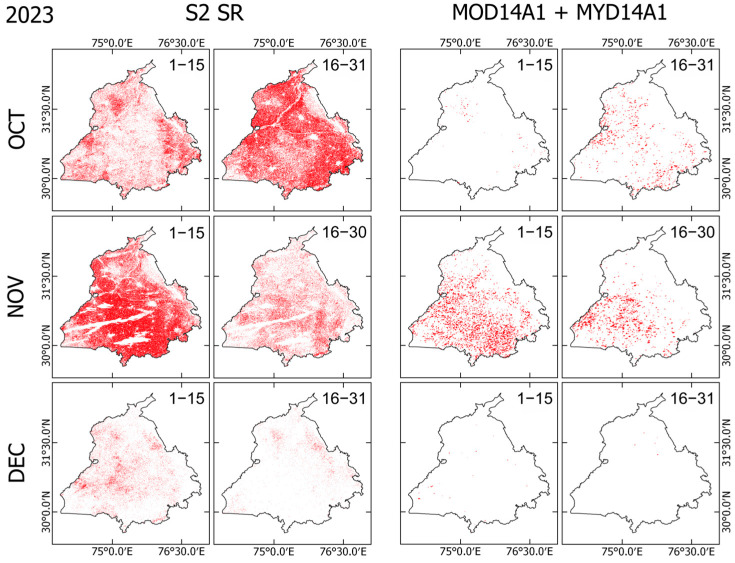
Comparison of newly detected burnt areas (BAs) from Sentinel-2 Surface Reflectance (S2 SR; columns 1–2) and MODIS (MOD14A1 + MYD14A1; columns 3–4) (as in Figure 7), but for the year 2023.

**Figure 9:**
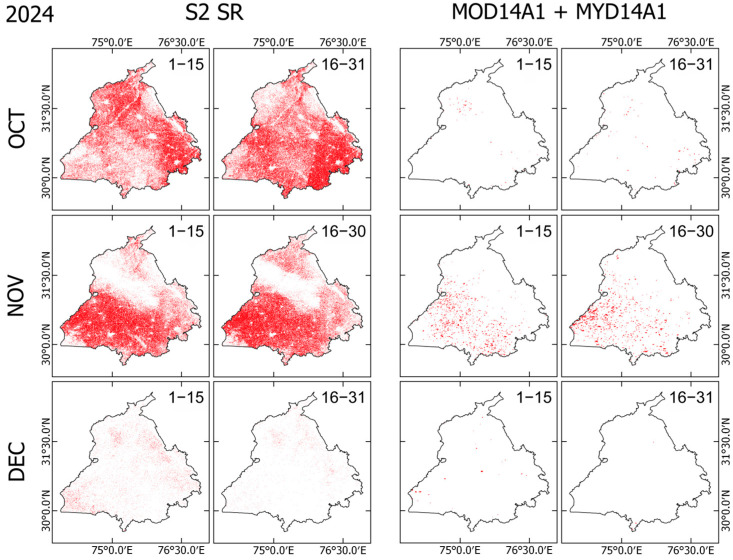
Comparison of newly detected burnt areas (BAs) from Sentinel-2 Surface Reflectance (S2 SR; columns 1–2) and MODIS (MOD14A1 + MYD14A1; columns 3–4) (as in Figure 7), but for the year 2024.

Comparison between Sentinel-2 SR and MODIS-based BA products shows considerable differences in both spatial extent and temporal coverage (Figure 7, Figure 8 and Figure 9). Across all years of 2022–2024, Sentinel-2 detected a markedly higher number of burnt pixels than MODIS, reflecting its higher spatial resolution (10–20 m vs. 1 km). This feature is apparent, especially in fragmented or narrow burnt fields, which are visible in Sentinel-2 but which are often completely absent from MODIS detections. Both sensors show the peak burning season in early November, aligning with agricultural residue burning after harvest. However, MODIS misses a large portion of the BA, especially during 1–15 November, when Sentinel-2 shows dense burn coverage across central and southern Punjab, whereas MODIS detections appear sparse.

MODIS detections are especially low at the end of November and December, which might result from persistent winter haze, cloud cover, and limited overpass timing. Sentinel-2, despite some cloud masking, still captures a much richer spatial extent of burning, particularly in the 1–15 December interval. MODIS fire pixels also show a tendency to cluster in central and southeastern Punjab, which potentially overemphasizes major clusters while missing small-scale fires. By contrast, Sentinel-2 reveals widespread and spatially continuous burn patterns, including smaller fires in northern and western regions. These results indicate that Sentinel-2 offers a much more detailed and complete picture of burning, whereas MODIS appears to underestimate both the extent and distribution of burnt areas systematically.

**Figure 10:**
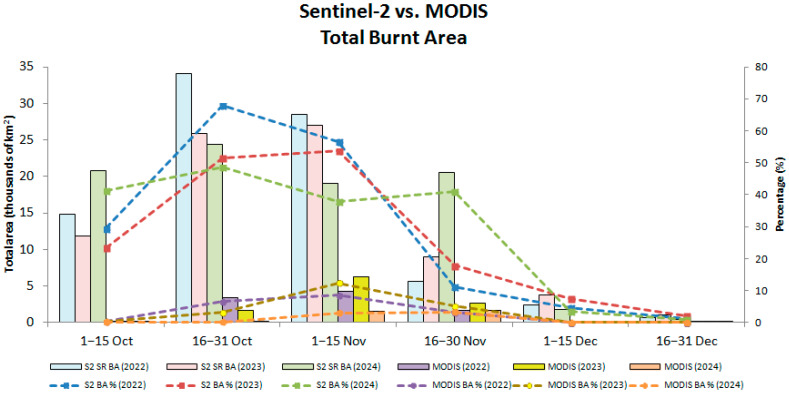
Total burnt areas (BAs) detected by Sentinel-2 Surface Reflectance (S2 SR) and MODIS during October–December in 2022–2024, shown as colored bar charts. BA percentages of corresponding periods for each sensor are presented as colored dashed lines.

As shown in Figure 10, across all three years, Sentinel-2 captured the highest BAs during the 16–31 October and 1–15 November windows, exceeding 34,000 km^2^, which represents more than 68% of the area of interest (AOI). By contrast, MODIS detects only a small fraction: generally below 7000 km^2^, even at its maximum, accounting for less than 15% of the AOI, and showing consistent underestimation of fire detection. Figure 10 also reveals clearer interannual differences detected by Sentinel-2, for instance, in 2024, 1–15 November appears to be lower than in 2022 and 2023, both in terms of area and percentage. In contrast, MODIS curves are flatter and less sensitive to year-to-year variation, likely masking subtle fire season intensity shifts.

### 3.4. Estimated PM_2.5_ Emissions from Sentinel-2Derived Burnt Areas

**Figure 11:**
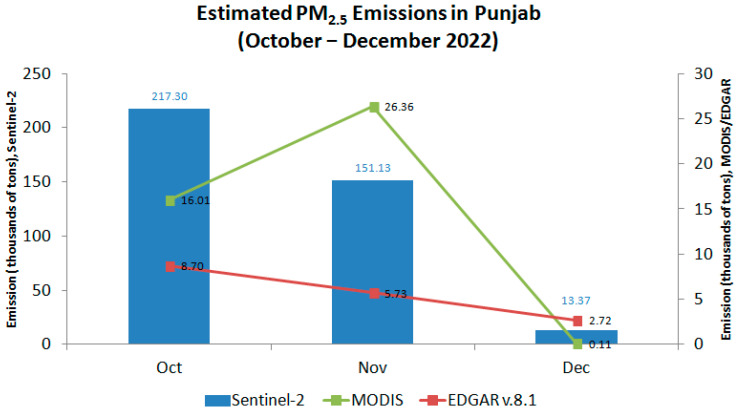
Chart diagram of PM_2.5_ emissions estimated by Sentinel-2-derived burnt area (BA) compared to MODIS BA and EDGARv.8.1 agricultural PM_2.5_ emissions during October–December 2022.

Based on burnt area data from Sentinel-2 and MODIS, as well as the EDGAR v.8.1 dataset, Figure 11 shows the estimated PM2.5 emissions in Punjab for the October–December 2022 period. EDGAR emissions are gridded monthly values from the agricultural sector at 0.1° × 0.1° resolution, whereas Sentinel-2 and MODIS emission estimates were calculated using two-weekly burnt area composites with assumptions for fuel load, combustion completeness, and emission factors.

Estimated Sentinel-2 emissions were 217.3 thousand tons in October, 151.1 thousand tons in November, and 13.4 thousand tons in December. By contrast, EDGAR presented much lower values for the same time period, with monthly totals of 8.7, 5.7, and 2.7 thousand tons, respectively. Both Sentinel-2’s and EDGAR’s derived emissions exhibit a steady declining trend during October–December, despite the marked magnitude differences. In contrast, estimated MODIS emissions exhibit an opposite trend between October and November, with the peak emission occurring in November, as opposed to October, as indicated by Sentinel-2 and EDGAR. The magnitude differences between MODIS and EDGAR emissions were notably smaller compared to Sentinel-2 (vs. EDGAR), with the maximum deviation being less than 21 thousand tons of PM_2.5_ in November. Another distinct feature is that MODIS emissions in December were notably lower than those of EDGAR, in contrast to the Sentinel-2 versus EDGAR comparison for the same period.

## 4. Discussion

For this study, after examining the spatiotemporal variability of BA during the post-monsoon fire season in Punjab for 2022–2024 using high-resolution Sentinel-2 observations, we validated the reliability of detection, compared the Sentinel-2-derived BA product with the MODIS BA product, estimated the PM_2.5_ emissions, and compared the findings with the global agricultural PM_2.5_ emission inventory of EDGAR v.8.1.

The dNBR histograms (Figure 3), burn severity maps (Figure 4), total BA, and mean severity time series (Figure 5) strongly indicated that, across all years, the burning activity started in early October, intensified gradually during late October, and finally reached a peak in early November before diminishing gradually in late November and December. This consistent pattern shows strong agreement with similar studies conducted earlier in northern areas of India [22,28,29,36]. Actually, 2024 shows a slight timing shift, with prolonged burning activity at the end of November. This minor discrepancy, however, does not undermine the overall consistency of the BA pattern highlighted earlier.

Another remarkable finding is the clear progression of BA originating in northern Punjab and shifting to central and southern Punjab during the 2022–2024 fire seasons. This striking feature confirms similar results obtained from earlier studies [30,44] and indicates the effect of harvest timing of paddy (rice) and wheat sowing in the state. Cultivation of earlier-maturing basmati varieties in the north, especially in the Majha region (Amritsar, Tarn Taran, Gurdaspur), leads to an earlier harvest time compared to those of the Malwa region in central and southern Punjab (Ludhiana, Sangrur, Bathinda, Patiala). Sentinel-2 observations captured this burnt area progression in sub-field scales of 10–20 m, which not only complements the findings of earlier studies but also offers versatile applications for monitoring and informing agricultural policy with much greater detail. This benefit is evident from validation obtained from ground photographs taken at different locations in Ludhiana on 30 October and 6 November 2022 (Figure 6), which demonstrate the capability of Sentinel-2 to differentiate burnt and unburnt fields with unprecedented accuracy.

Comparison between Sentinel-2 and MODIS BA products (Figure 7, Figure 8, Figure 9 and Figure 10) reveals the benefits of Sentinel-2 observations for providing clearer pictures of BA spatiotemporal variability during the fire season in Punjab. The higher spatial resolution of Sentinel-2 enables the detection of fragmented areas, leading to a considerably higher total BA estimate, reaching nearly 70% of the state’s total area during the peak of fire season, compared to MODIS detection, which rarely exceeded 10% of Punjab’s total area. The consistent underestimation of BA estimates by MODIS confirms findings obtained from earlier studies [25,45]. This detection discrepancy between Sentinel-2 and MODIS might derive from differences in detection strategies between those of the two sensors. On the one hand, MODIS uses a contextual algorithm [46,47] which exploits the strong emission of mid-infrared radiation from fires [48,49]. The active fire might be undetected if the fire were too small or too ‘cool’ to be detected in the 1 km^2^ MODIS footprint. Furthermore, extensive cloud cover, tree canopy, or heavy smoke during the peak of the stubble burning period might obscure the fire completely, which makes the likelihood of detection very low [45,50]. On the other hand, Sentinel-2 exploits the spectral reflectance characteristics of the Earth’s surface rather than measuring the fire radiative power directly, thereby enabling the detection of burning scars, even in the absence of an active fire.

Despite the benefits of Sentinel-2’s high spatial resolution, Figure 8 also reveals sensor limitations under persistent cloud or fog cover. This limitation is evident during 1–15 November 2023, when Sentinel-2 detected considerably less BA coverage than in either 2022 or 2024. By stark contrast, MODIS consistently detected a dense fire cluster in central and southern Punjab during this period. Given the Sentinel-2 5-day revisiting frequency and dependence on cloud-free optical observation, the extensive haze, fog, or cloud cover might have obscured the sensor’s detection of burn scars, leading to underestimation of burnt pixels. MODIS, with twice-daily overpasses and an active fire detection algorithm, is often less affected by thin clouds or haze, which might explain its stable fire detection during the period.

A large discrepancy is also evident, as shown in Figure 11, where the Sentinel-2-derived PM_2.5_ emissions during October–December 2022 were much higher than the EDGAR v.8.1 emission inventory, although exhibiting a similar trend. Our PM_2.5_ emission estimates depend linearly on three key parameters: effective fuel load (B), burning efficiency (BE), and PM_2.5_ emission factor (EF). To assess robustness, we performed a sensitivity analysis using B = 0.61 kg m^−2^ (midpoint of the cited range), BE ∈ [0.5, 0.9], and EF ∈ [4.2, 20.7] g kg^−1^ (literature range), as shown in Table 2. For the peak burnt area observed (~35,000 km^2^), the resulting PM_2.5_ estimates range between ~4.5 × 10^4^ and ~4.0 × 10^5^ tons for the biweekly period, illustrating that reasonable variations in BE and EF can change totals by an order of magnitude. Even after adopting conservative parameter values, Sentinel-2-based estimates remain substantially higher than EDGAR v8.1 for the same period.

This marked difference likely originates from EDGAR’s activity data and gridding approach, which do not capture the fine-scale, high-frequency stubble burning that Sentinel-2 resolves. The EDGAR database uses annual data and a consistent EF approach rather than the direct remote sensing approach used for this study. Although the homogeneity of the method is a key point of EDGAR, it might engender uncertainties when similar emission sources are aggregated [51]. For agricultural waste burning in particular, EDGAR estimated the fraction for crop residues removed and/or burnt mainly based on statistical data and official country reporting [52], instead of using the latest in situ or remote sensing observations. Therefore, the uncertainty is regarded as very high: 50–100% [53]. Nevertheless, the same uncertainty issue applies for emissions estimated using the dNBR method. Although providing detection of the burnt area in high resolution, the estimated emissions depended strongly on assumptions used to determine the PM_2.5_ emissions, such as the biomass loading and burning efficiency, which might differ for each region of interest. This limitation suggests that future studies particularly addressing crop residue burning parameters obtained from the latest field measurements over Punjab are necessary to achieve more accurate estimates of PM_2.5_ emissions from the region.

In contrast, MODIS-derived emissions displayed a different temporal evolution, with the peak occurring in November rather than October, as indicated by Sentinel-2 and EDGAR, which might arise from MODIS’s coarser spatial resolution and detection threshold. Furthermore, the magnitude difference between MODIS and EDGAR was notably smaller than that between Sentinel-2 and EDGAR, suggesting that MODIS estimates are closer to the conservative, inventory-driven EDGAR values, albeit at the expense of under-detecting smaller or fragmented burns. Another distinct feature emerged in December, when MODIS emissions fell below EDGAR, whereas Sentinel-2 remained higher, underscoring Sentinel-2’s sensitivity to residual or scattered burning activities that may be overlooked by MODIS. Overall, these findings highlight the enhanced capability of Sentinel-2 to capture fine-scale burned areas and associated emissions while also emphasizing the tendency of MODIS and EDGAR to underestimate short-term or localized fire activity.

One limitation of our approach is related to the reburning scenario, where a pixel that has been severely burnt in a previous time step may experience a subsequent fire of smaller extent. In such cases, the dNBR signal may still remain positive but close to the threshold value, leading to possible underestimation or ambiguity in detecting reburned pixels. Although the applied 0.1 dNBR threshold is consistent with previous studies and helps minimize false positives, this choice may exclude some marginal or low-intensity burn signals. As a result, our emission estimates should be interpreted with this inherent uncertainty in mind.

Another source of uncertainty arises from the treatment of effective fuel load to estimate PM_2.5_ emission. While our approach incorporates biomass loading and burning efficiency, it does not explicitly capture temporal variations in fuel availability driven by post-fire vegetation regrowth. Tracking such changes is challenging, as fuel load datasets with sufficient spatial and temporal detail are generally unavailable, and most studies therefore rely on literature-based values. In regions affected by recurrent fires, rapid vegetation recovery may alter the effective fuel load between successive events, which in turn could influence subsequent emission estimates. Although our framework does not explicitly address this process, the use of pixel-specific dNBR and burned area provides a reliable first-order estimate of fire severity and extent, thereby reducing the risk of systematic bias. Future studies could further improve emission estimates by coupling burned area observations with dynamic vegetation or land cover models to account for fuel recovery between fire cycles.

Despite these limitations, the use of Sentinel-2 data with a pixel-specific dNBR approach provides a consistent and high-resolution estimate of burnt area and associated PM_2.5_ emissions. This complements coarser-resolution products such as MODIS and inventory-based datasets like EDGAR, and together they offer a more complete picture of regional fire emissions. Consequently, our study contributes to narrowing the gap between satellite-derived observations and bottom-up inventories, thereby supporting more accurate assessments of biomass burning impacts on air quality.

## 5. Conclusions

Results of this study demonstrate the capability of Sentinel-2 high-resolution observation to detect stubble-burning activity and estimate PM_2.5_ emission in Punjab, India. By analyzing the spatiotemporal variability of the burnt areas during October–December for three consecutive years, we found that Sentinel-2 consistently captures burn scars with greater spatial detail than that provided by coarser resolution products such as MODIS. The two-weekly composites of Sentinel-2 imagery enable us to ascertain more details of the timing of burning events and to identify the phases of burning, which align with regional harvesting cycles and agricultural practices prevailing in Punjab.

Visual validation using ground photographs confirmed the accuracy of Sentinel-2-derived burn severity. Subsequent comparison with MODIS revealed that MODIS underestimated the extent and variability of the burnt areas, especially during the peak of fire season. Despite limitations during extensive cloud cover, the overall performance of Sentinel-2 attests to its versatility for regional fire monitoring and for informing agricultural policy in the region.

Although exhibiting a similar trend, the estimated PM_2.5_ emissions found from Sentinel-2-derived burnt areas were markedly higher than those found by EDGAR v.8.1. This discrepancy might derive from differences in their respective methodologies: Sentinel-2 provides a direct estimate from remote sensing observations, whereas EDGAR emissions were acquired primarily from global statistics. This finding highlights the importance of assimilation between remote sensing and statistical in situ observation to improve accuracy for future studies.

Finally, the utilization of cloud-based platforms such as Google Earth Engine has been shown to be fundamentally necessary for developing high-resolution fire emission inventories to support air quality monitoring, modeling, environmental policy, and land management strategies.

## Figures and Tables

**Table 1:** Burn severity values calculated from dNBR.

Severity Level	dNBR Range (Scaled by 10^3^)
Unburnt	Less than 100
Low severity	+100–+269
Moderate severity (low)	+270–+439
Moderate severity (high)	+440–+659
High severity	+660–+1300

**Table 2:** Sensitivity of Sentinel-2-derived PM_2_._5_ emission estimates (thousand tons) to variations in burning efficiency (BE) and emission factor (EF) for a peak burnt area of ~35,000 km^2^. Calculations assume a biomass loading of 0.61 kg m^−2^ (midpoint of the 0.42–0.80 kg m^−2^ range).

Burning Efficiency	Emission Factor (g kg^−1^)	PM_2.5_ Emission (Thousand Tons)
0.5	0.0042	44.84
0.5	0.0091	97.14
0.5	0.0207	220.97
0.8	0.0042	71.74
0.8	0.0091	155.43
0.8	0.0207	353.56
0.9	0.0042	80.70
0.9	0.0091	174.86
0.9	0.0207	397.75

## Data Availability

The original data presented in the study are openly available in Google Earth Engine Data Catalog (https://developers.google.com/earth-engine/datasets, accessed on 1 May 2025). This includes Sentinel-2: Surface Reflectivity (“COPERNICUS/S2_SR_HARMONIZED”), Sentinel-2: Cloud Probability (“COPERNICUS/S2_CLOUD_PROBABILITY”), ESA WorldCover 10 m v200 (“ESA/WorldCover/v200”), MOD14A1.061: Terra Thermal Anomalies & Fire Daily Global 1 km and MYD14A1.061: Aqua Thermal Anomalies & Fire Daily Global 1 km (“MODIS/061/MOD14A1” and “MODIS/061/MYD14A1”, respectively). The EDGAR Global Agricultural PM_2.5_ v.8.1 emission dataset is openly available in EDGAR Global Emission Datasets (https://edgar.jrc.ec.europa.eu/dataset_ap81, accessed on 1 June 2025). Ground photos are obtained from the AAKASH project campaign in Punjab during October-November 2022.

## Abbreviations

The following abbreviations are used in this manuscript:

PM	Particulate Matter
NDVI	Normalized Difference Vegetation Index
NDWI	Normalized Difference Water Index
NBR	Normalized Burn Ratio
dNBR	Delta Normalized Burn Ratio
MODIS	Moderate Resolution Imaging Spectrometer
VIIRS	Visible Infrared Imaging Radiometer Suite
FHS	Fire Hotspot
BA	Burnt Area
GEE	Google Earth Engine
S2 SR	Sentinel-2 Surface Reflectance
SCL	Scene Classification Layer
EDGAR	Emissions Database for Global Atmospheric Research
AD	Activity Data
NIR	Near-Infrared
SWIR	Short Wave Infrared
EF	Emission Factor
AOI	Area of Interest
